# Therapeutic Delivery of Soluble Fractalkine Ameliorates Vascular Dysfunction in the Diabetic Retina

**DOI:** 10.3390/ijms25031727

**Published:** 2024-01-31

**Authors:** Derek Rodriguez, Kaira A. Church, Chelsea T. Smith, Difernando Vanegas, Sandra M. Cardona, Isabel A. Muzzio, Kevin R. Nash, Astrid E. Cardona

**Affiliations:** 1 Department of Molecular Microbiology and Immunology, The University of Texas at San Antonio, San Antonio, TX 78249, USA; derek.rodriguez@utsa.edu (D.R.); kachurch@mdanderson.org (K.A.C.); chelsea.smith@my.utsa.edu (C.T.S.); difernando.vanegas@utsa.edu (D.V.); sandra.cardona@utsa.edu (S.M.C.); 2 Department of Psychological and Brain Sciences, University of Iowa, Iowa City, IA 52242, USA; isabel-muzzio@uiowa.edu; 3 Department of Molecular Pharmacology and Physiology, University of South Florida, Tampa, FL 33612, USA; nash@usf.edu

**Keywords:** microglia, macrophages, fractalkine, vasculature, diabetic retinopathy, gene therapy

## Abstract

Diabetic retinopathy (DR)-associated vision loss is a devastating disease affecting the working-age population. Retinal pathology is due to leakage of serum components into retinal tissues, activation of resident phagocytes (microglia), and vascular and neuronal damage. While short-term interventions are available, they do not revert visual function or halt disease progression. The impact of microglial inflammatory responses on the neurovascular unit remains unknown. In this study, we characterized microglia–vascular interactions in an experimental model of DR. Early diabetes presents activated retinal microglia, vascular permeability, and vascular abnormalities coupled with vascular tortuosity and diminished astrocyte and endothelial cell-associated tight-junction (TJ) and gap-junction (GJ) proteins. Microglia exclusively bind to the neuronal-derived chemokine fractalkine (FKN) via the CX3CR1 receptor to ameliorate microglial activation. Using neuron-specific recombinant adeno-associated viruses (rAAVs), we therapeutically overexpressed soluble (sFKN) or membrane-bound (mFKN) FKN using intra-vitreal delivery at the onset of diabetes. This study highlights the neuroprotective role of rAAV-sFKN, reducing microglial activation, vascular tortuosity, fibrin(ogen) deposition, and astrogliosis and supporting the maintenance of the GJ connexin-43 (Cx43) and TJ zonula occludens-1 (ZO-1) molecules. The results also show that microglia–vascular interactions influence the vascular width upon administration of rAAV-sFKN and rAAV-mFKN. Administration of rAAV-sFKN improved visual function without affecting peripheral immune responses. These findings suggest that overexpression of rAAV-sFKN can mitigate vascular abnormalities by promoting glia–neural signaling. sFKN gene therapy is a promising translational approach to reverse vision loss driven by vascular dysfunction.

## 1. Introduction

Diabetes is characterized by a persistent hyperglycemic microenvironment, leading to severe health complications such as blindness. Diabetic retinopathy (DR) is an ocular impairment affecting nearly 10 million individuals in the United States [1,2,3]. This number is expected to increase due to the surge in obesity-associated diabetes. DR is marked by retinal vascular and neuronal damage and immune cell activation propagated by local and systemic inflammation associated with recurrent infections in patients with diabetes. Existing treatments for DR fall short, yielding favorable short-term results but lacking the ability to halt or reverse the progression of the disease. Additionally, there are no therapies that limit or reverse disease progression.

The retina is a highly metabolically active organ housing three distinct types of glial cells: Müller cells, astrocytes, and microglia. Müller cells extend across all retinal layers and play a vital role in regulating neuronal metabolism by managing glutamate uptake, maintaining ionic balance, releasing trophic factors, and controlling vasculature. Astrocytes are primarily located in the nerve fiber layer and ganglion cell layer, serving similar functions to Müller cells while also overseeing the integrity of the blood-brain barrier and retinal capillaries. Microglia, the innate resident macrophages of the central nervous system, are responsible for controlling the spread of harmful insults, playing a critical role in both injury recovery and the progression of inflammation within the retina. The retinal vascular networks are crucial in supporting neuronal and glial cells [4]. Arising from the optic disk, arterioles follow a sequential branching pattern as they traverse into retinal capillaries. These capillaries form the superficial vascular plexus and then penetrate deep into the layers of the retina before connecting back to the venules, completing the vascular circuit. Within the vascular branches, endothelial cells (ECs) and astrocytes are essential to vascular formation, integrity, and homeostasis [5]. Connexin-43 (Cx43) gap junctions (GJs) facilitate communication in ECs and are selectively downregulated in the early stages of DR, leading to disrupted vasomotor responses and ineffective blood flow [5]. Cx43 GJs are colocalized and interact closely with the tight junction (TJ) proteins claudin 5, occludin, and zonula occludens-1 (ZO-1). These proteins play a role in preserving and maintaining barrier properties of ECs and end-foot processes of astrocytes [6,7,8,9]. Additional studies have shown that neurons influence the vascular profile through calcium-dependent cross-talk with immune and Müller glial cells within the retina, which are established players contributing to vascular function [10,11].

Microglia are known to engage in synaptic pruning, immune surveillance, phagocytosis, and recruitment of other immune cells through the production of cytokines. Additionally, they have been noted to initiate the breakdown of the blood-retinal barrier (BRB) through the production of IL-1β and TNF-α. Hyperactivation of microglia damages the BRB, as indicated by the increased leakage of the coagulating serum protein fibrin(ogen), which is often observed at elevated levels in diabetic patients. We discovered that microglia play a key role in regulating the neurovascular unit through its receptor CX3CR1. Fibrin(ogen) depletion via systemic treatment of ancrod dampens the density of microglial lesions and pro-inflammatory cytokines, alleviating vascular damage in diabetic mice lacking CX3CR1 (CX3CR1^KO^) [12]. Depletion of microglia using the CSF-1R antagonist PLX-5622 protects diabetic retinas from angiogenesis in experimental DR [13]. Administration of the recombinant chemokine fractalkine (FKN; CX3CL1) to fractalkine-knockout (FKN^KO^) mice reduces perivascular clustering and fibrin(ogen) deposition and vascular permeability [14]. These studies suggest that the FKN/CX3CR1 signaling axis serves as a regulator of microglia-mediated vascular damage.

FKN is a unique anti-inflammatory chemokine expressed by neurons and endothelial cells. Membrane-bound FKN (mFKN) acts as an adhesion molecule and is constitutively cleaved to release the soluble (sFKN) chemokine isoform. A disintegrin metalloproteinase (ADAM) 10/17, tumor necrosis factor-alpha (TNF-α)–converting enzyme (TACE), and cathepsin S contribute to sFKN release from its mFKN form. Diabetic FKN^KO^ retinas show enhanced microglia activation, neuronal loss, and significant fibrin(ogen) deposition, mirroring the phenotype of mice expressing the human polymorphic variants of the CX3CR1 gene (hCX3CR1^I249/M280^) and CX3CR1^KO^ DR models [14]. Intra-vitreal administration of recombinant adeno-associated virus (rAAV)-sFKN in FKN^KO^ mice before the induction of diabetes reduces fibrin(ogen) deposition, microgliosis, inflammation, and vascular damage compared to rAAV-mFKN [15]. Therefore, this study tests the hypothesis that sFKN overexpression in wild-type mice confers vascular protection and enhances visual acuity. Results show that administration of rAAV-sFKN but not rAAV-mFKN supports vascular integrity and improved visual function, without affecting peripheral immune responses. These findings suggest that overexpression of rAAV-sFKN can mitigate vascular abnormalities by promoting glia–neural signaling.

## 2. Results

### 2.1. Neuronal Densities in Retinas Treated with sFKN or mFKN Are Comparable to Non-Diabetic Groups

**Figure 1:**
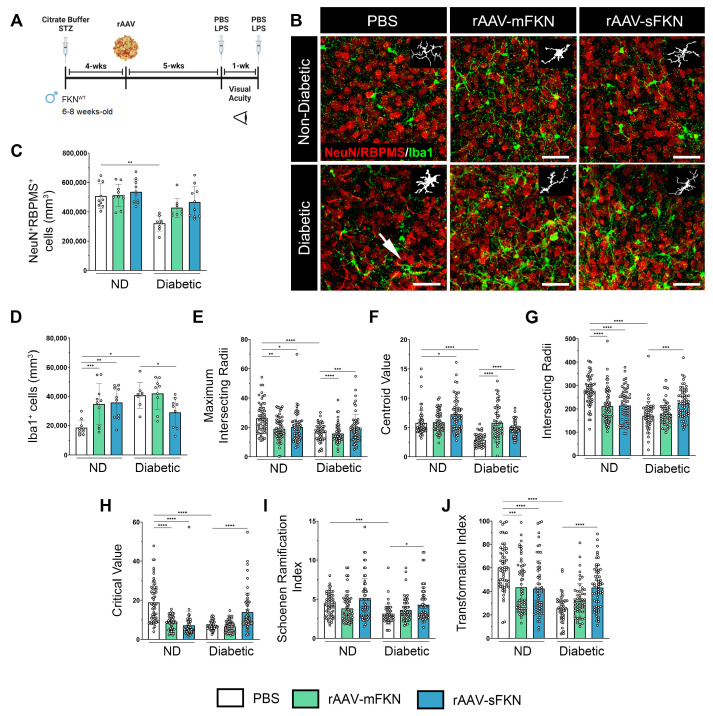
rAAV-sFKN reduces morphological microglial activation, increasing their ramified processes in the diabetic retina. (**A**) Experimental design to investigate the effects of intra-vitreal delivery of rAAV-mFKN or rAAV-sFKN to the retina. (**B**) Confocal images of retinal tissues stained for NeuN (red) and Iba1 (green), with representation of cellular tracing for transformation index and Sholl analyses (*Inset*) (Scale bar; 50 µm). (**C**,**D**) Quantification of retinal immunofluorescence analysis of NeuN^+^ cells/mm^3^ (**C**) and Iba1^+^ cells/mm^3^ (**D**). Data are shown as the mean ± SD, *n* = 8–10 mice per group, where each dot represents an individual mouse. (**E**–**J**) Microglial morphology was assessed by calculating the maximum intersecting radii (**E**), centroid value (**F**), intersecting radii (**G**), critical value (**H**), Schoenen ramification index (**I**), and transformation index (**J**). Each dot represents an individual microglia, *n* = 48–60 per group. Data are shown as the mean ± SD. * *p* < 0.05, ** *p* < 0.01, *** *p* < 0.001, and **** *p* < 0.0001 using one-way ANOVA, Kruskal–Wallis test.

rAAVs expressing mFKN or sFKN were shown to reconstitute retinal FKN levels in FKN^KO^ to levels comparable to WT mice (Figure S1A). To assess the role of mFKN and sFKN on neurodegeneration and microgliosis after diabetes induction, retinal tissues were stained and imaged to analyze NeuN^+^ neuronal cell bodies and Iba1^+^ microglia (Figure 1A,B). Analysis of retinal tissues after 10 weeks of hyperglycemia revealed a decrease in NeuN^+^ neuronal densities in diabetic mice (320,952 ± 52,803) in comparison to the non-diabetic (ND) controls (507,612 ± 84,163, one-way ANOVA *p* < 0.001). Groups treated with sFKN or mFKN showed neuronal densities comparable to those from non-diabetic controls, suggesting that FKN exerts a neuroprotective effect (Figure 1C). PBS-treated diabetic mice showed an increase in Iba1^+^ cells compared to their ND counterparts (40,844 ± 8810 and 18,627 ± 5177, one-way ANOVA *p* < 0.01). Interestingly, we observed elevated microglial densities in ND mice treated with mFKN (34,741 ± 14,043, one-way ANOVA *p* < 0.001) or sFKN (35,844 ± 11,154 *p* < 0.01). In diabetic mice, sFKN (29,201 ± 10,111 Student’s *t* test with Welch’s correction *p* < 0.05) appeared to decrease the number of Iba1^+^ cells compared to the diabetic PBS-treated group (Figure 1D). Overall, microglial densities were not altered in the diabetic groups in response to sFKN or mFKN treatment.

### 2.2. rAAV-sFKN Administration Minimizes Microglial Hyperreactivity during Diabetes

We next compared the degree of microglial activation using quantitative methods to assess their morphology and degree of ramification. Using Iba1^+^ immunofluorescence, we analyzed microglial densities, microglial activation through Sholl analysis, the Schoenen ramification index (RI), and the transformation index (TI). The automated Sholl analysis plot shows the branching pattern of microglia (Figure S1B,C). Additional parameters of the Sholl analysis demonstrated that rAAV-sFKN treatment significantly increased the maximum intersection radii (19.25 ± 9.59, one-way ANOVA *p* < 0.001), centroid value (4.66 ± 1.27, one-way ANOVA *p* < 0.0001), intersecting radii (225.0 ± 62.77, one-way ANOVA *p* < 0.001), and critical value (13.95 ± 9.82, one-way ANOVA *p* < 0.0001) in diabetic mice compared to PBS-treated controls (18.29 ± 5.93, 2.90 ± 1.03, 170.60 ± 59.94, 7.05 ± 2.20, respectively) (Figure 1E–H). Treatment with rAAV-mFKN and rAAV-sFKN in ND conditions displayed significant increases in microglial morphological activation compared to PBS-treated mice. Similarly, the RI revealed that microglial processes were less branched in PBS-treated diabetic mice (3.08 ± 1.28) when compared to cells in the PBS-treated ND mice (4.31 ± 1.44, one-way ANOVA *p* < 0.001). Microglia are significantly re-ramified with the administration of rAAV-sFKN (4.3 ± 2.18, one-way ANOVA *p* < 0.05) during diabetes when compared to their PBS-treated diabetic counterparts (Figure 1I). From the Sholl analysis, we extracted data to determine the TI of microglia by automatically outlining the cell from our previous morphological comparisons. Retinal tissues illustrated a distinctive decrease in the TI of PBS-treated diabetic mice (25.50 ± 11.44) compared to their ND counterparts (60.28 ± 19.84, one-way ANOVA *p* < 0.0001). Administration of vectors expressing mFKN (43.52 ± 21.51, one-way ANOVA *p* < 0.001) or sFKN (42.47 ± 23.83, one-way ANOVA *p* < 0.0001) elicited a significant decrease in their TI compared to PBS-treated ND mice. rAAV-sFKN treatment in diabetic mice (43.37 ± 18.23, one-way ANOVA *p* < 0.0001) significantly elevated the microglial TI profile compared to PBS-treated diabetic mice (Figure 1J). These results show that microglial morphological activation was downregulated by sFKN, which was confirmed by multifactorial morphological analysis.

Flow cytometric analysis of brain and spinal cord tissues revealed that microglia in diabetic PBS-treated animals (93.04 ± 5.12) expressed significantly lower levels of a homeostatic marker (CD11b^+^CD45^Lo^P2RY12^+^) compared to PBS-treated ND controls (99.93 ± 0.06, one-way ANOVA *p* < 0.0001) (Figure S1D–H). Treatment with rAAV-sFKN (98.88 ± 0.56, one-way ANOVA *p* < 0.001) during diabetes showed a significant increase in homeostatic microglia compared to PBS-treated diabetic mice (Figure S1G). These results are consistent with the microglial phenotype observed by Iba1^+^ immunofluorescence, Sholl analysis, the RI, and the TI. These findings indicate that rAAVs can spread within the CNS, resulting in the overexpression of FKN. This overexpression, in turn, influences microglial morphology, causing a shift towards a more branched and ramified cellular shape.

We assessed the frequencies of innate myeloid cells in peripheral blood to determine whether rAAVs altered systemic immune responses in diabetic animals. We found that overexpression of sFKN or mFKN via rAAV treatment in diabetic mice did not alter peripheral blood frequencies of neutrophils (CD45^Hi^CD11b^+^SSC^Hi^) or conventional (CD45^Hi^CD11b^−^CD11c^+^) or myeloid-derived (CD45^Hi^CD11b^+^CD11c^+^) dendritic cells (DCs) when compared to PBS-treated ND controls (Figure S2). Changes were detected in diabetic versus non-diabetic groups when comparing non-classical (CD45^Hi^CD11b^+^Ly6C^−^) and classical (CD45^Hi^CD11b^+^Ly6C^+^) monocytes. In the diabetic groups, rAAV did not induce changes in the myeloid cell populations. These data indicate that intra-vitreal administration of rAAV-sFKN or rAAV-mFKN does not alter the systemic innate profile during diabetes.

### 2.3. sFKN Alleviates Aberrant Vascular Permeability in the Diabetic Retina

**Figure 2:**
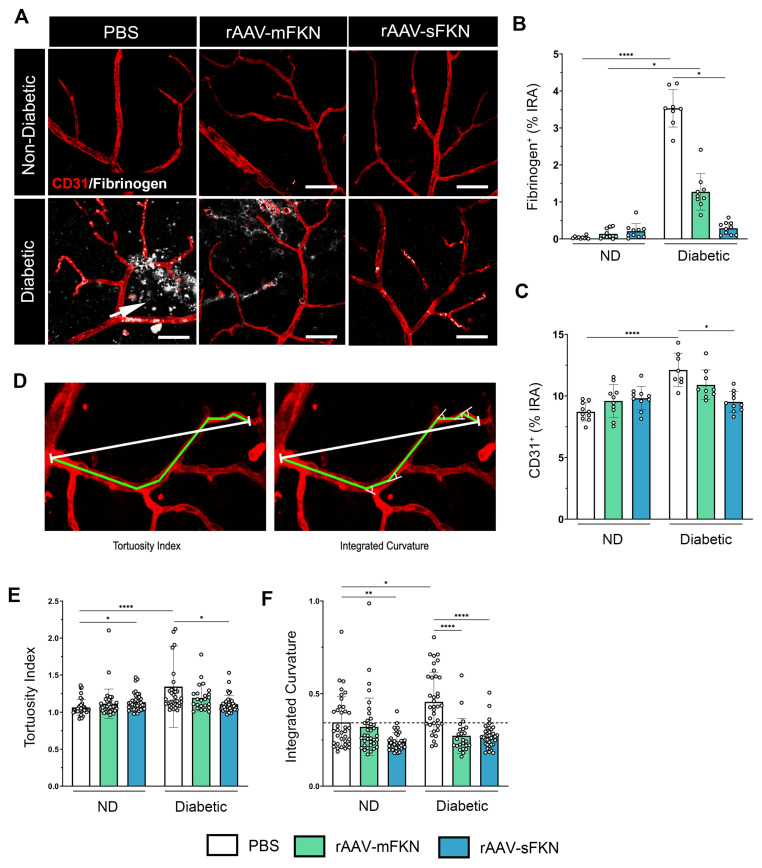
sFKN gene therapy reduces fibrinogen deposition and vascular abnormalities in the diabetic retina. (**A**) Confocal images of retinal tissues stained for CD31 (red) and fibrinogen (white), arrow points to extravasated fibrinogen from the blood vessels. (**B**,**C**) Quantification of retinal immunofluorescence analysis of the fibrinogen^+^ percent immunoreactive area (% IRA) (**B**) and CD31^+^ immunoreactive area (% IRA) (**C**). Data are shown as the mean ± SD, *n* = 8–10 mice per group, where each dot represents an individual mouse. (**D**) The tortuosity index (ToI) was obtained by dividing the length of a segmented line following the vessel (green line) by the distance between the start and end point (white line) (**E**). The sum of the angles between the line segments was divided by the distance between the start and end point and used as an approximation of the integrated curvature (IC) (**F**). Data are shown as the mean ± SD, *n* = 96–120 per group, where each dot represents an individual measured vessel (scale bar; 50 µm). * *p* < 0.05, ** *p* < 0.01, and **** *p* < 0.0001 using one-way ANOVA, Kruskal–Wallis test.

Next, we evaluated vascular abnormalities in the diabetic retina by measuring fibrin(ogen) extravasation from the vasculature and anomalous neovascularization utilizing high-resolution confocal imaging (Figure 2A). Fibrinogen aggregates were visualized near areas of ruptured endothelium, which were significantly upregulated in PBS-treated diabetic mice (3.53 ± 0.50) compared to PBS-treated ND mice (0.03 ± 0.03, one-way ANOVA *p* < 0.0001). Similarly, mFKN-treated diabetic mice showed increased fibrin(ogen) deposition compared to their respective controls (1.27 ± 0.49 and 0.14 ± 0.13, one-way ANOVA *p* < 0.05). sFKN-treated, but not mFKN-treated groups (0.28 ± 0.16 and 1.27 ± 0.49, respectively), displayed less fibrin(ogen) extravasation compared to the PBS-treated diabetic mice (one-way ANOVA *p* < 0.05) (Figure 2B). Additionally, PBS-treated ND mice (8.71 ± 0.745) had a significantly lower CD31^+^ immunoreactive area compared to their diabetic counterparts (12.11 ± 1.36, one-way ANOVA *p* < 0.0001) (Figure 2C), indicative of less vascular damage. Notably, rAAV-sFKN (9.52 ± 0.82, one-way ANOVA *p* < 0.05) administration was able to significantly decrease the CD31^+^ immunoreactive area in diabetes compared to the PBS-treated group.

To further assess the level of EC irregularity, we performed an image analysis to determine the ToI (Figure 2D). PBS-treated ND mice (1.06 ± 0.109) showed decreased vascular contortions compared to PBS-treated diabetic mice (1.34 ± 0.54, one-way ANOVA *p* < 0.0001). Administration of rAAV-sFKN (1.10 ± 0.11), but not rAAV-mFKN (1.19 ± 0.17), significantly decreased the vascular ToI compared to PBS-treated diabetic mice (Figure 2E). To further assess the curvature of the tortuous ECs, we measured EC angularity via the integrated curvature index. EC curvature was significantly increased in PBS-treated diabetic mice (0.45 ± 0.15) compared to their ND counterparts (0.34 ± 0.14, one-way ANOVA *p* < 0.05). Vessel angularity was ameliorated in diabetic mice with rAAV expressing mFKN (0.27 ± 0.09) or sFKN (0.27 ± 0.06) compared to the PBS-treated diabetic mice (one-way ANOVA *p* < 0.0001) (Figure 2F). These results show that sFKN supports vascular remodeling and retinal EC linearity.

### 2.4. Administration of rAAVs Expressing mFKN or sFKN Increases Microglia–Vascular Interactions Influencing Capillary Constriction

**Figure 3:**
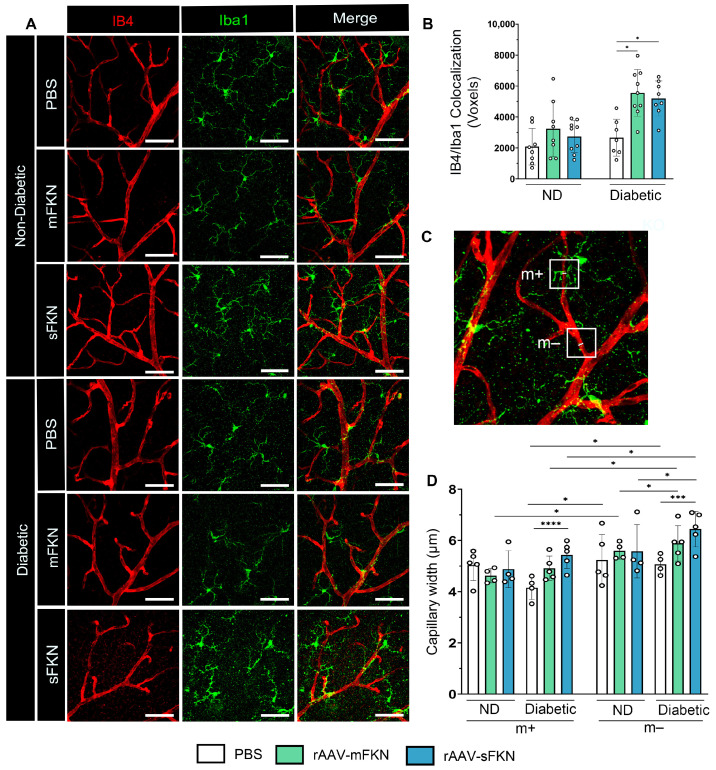
rAAV expression of FKN isoforms increases microglial vascular interactions, contributing to vascular constriction. (**A**) Confocal images of retinal tissues stained for IB4 (red) and Iba1 (green) (Scale bar; 50 µm). (**B**) Double immunofluorescence for IB4 and Iba1 using Imaris software. The number of double-positive colocalized pixels (Voxels) for IB4/Iba1. Data are shown as the mean ± SD, *n* = 7–9 mice per group, where each dot represents an individual mouse. (**C**–**D**) Capillary diameter was quantified by using Image J software on digital images (**C**). Analysis of vascular diameter at sites with and without microglial contact (**D**). Data are shown as the mean ± SD, *n* = 4–5 capillary regions, where each dot represents one mouse averaging two capillary regions per image (6 images total per mouse) with microglia contact (m+) and without microglia contact (m−) (Scale bar; 50 µm). * *p* < 0.05, *** *p* < 0.001, and **** *p* < 0.0001 using one-way ANOVA, Kruskal–Wallis test.

To confirm the mechanism by which microglia-mediated vasculo-protection occurs with FKN overexpression, we investigated the interaction between Iba1^+^ microglia and IB4^+^ ECs upon administration of mFKN or sFKN. High-resolution confocal imaging was used to examine the interactions between glial cells and the vascular system within the superficial vascular plexus in both ND and diabetic retinas using a colocalization analysis (Figure 3A). The results showed a significant increase in vascular-associated microglia in diabetic mice that received mFKN (5559 ± 1512) or sFKN (5195 ± 1136) compared to PBS-treated controls (2666 ± 1195, one-way ANOVA *p* < 0.05) (Figure 3B). We examined if vasoconstriction associated with microglia in the vessels was regulated by overexpression of FKN via rAAVs (Figure 3C). In response to overexpression of mFKN (4.91 ± 0.47) or sFKN (5.42 ± 0.52), we found a decrease in capillary diameter in regions associated with microglial processes (m+) compared to vascular regions that were distant from microglia (m−) (Figure 3D). Diabetic vascular regions without microglia contact had increased capillary widths compared to regions with microglial contact (mFKN: 4.91 ± 0.47 and sFKN: 5.42 ± 0.52 with microglia contact vs. mFKN: 5.88 ± 0.69 and sFKN: 6.44 ± 0.69 without microglia contact, one-way ANOVA *p* < 0.05). These results suggest that rAAV therapy not only increases vascular-associated microglia but also influences EC diameter.

### 2.5. sFKN Minimizes Reactive Astrocytes and Reduces Tight- and Gap-Junction Expression during Diabetes

**Figure 4:**
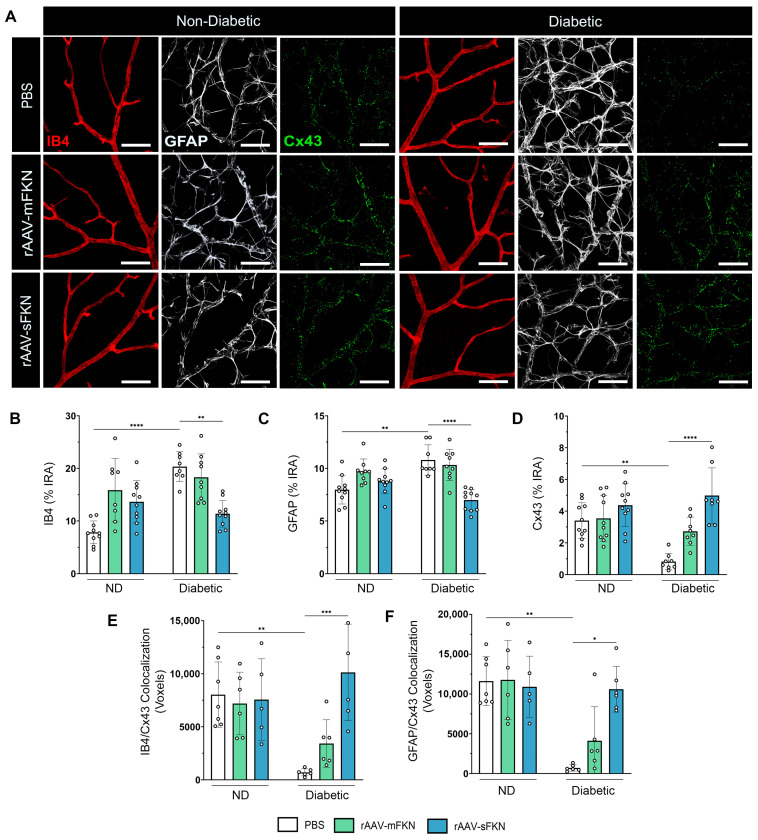
sFKN gene therapy protects vascular gap-junction loss in the diabetic retina. (**A**) Confocal images of retinal tissues stained for IB4 (red), GFAP (white), and Cx43 (green). (**B**–**D**) Quantification of retinal immunofluorescence analysis of the IB4^+^ immunoreactive area (% IRA) (**B**), the GFAP^+^ immunoreactive area (% IRA) (**C**), and the Cx43^+^ immunoreactive area (% IRA) (**D**). (**E**,**F**) The number of double-positive colocalized pixels (voxels) for IB4/Cx43 (**E**) and GFAP/Cx43 (**F**). Data are shown as the mean ± SD, *n* = 6–10 mice per group, where each dot represents an individual mouse. * *p* < 0.05, ** *p* < 0.01, *** *p* < 0.001, and **** *p* < 0.0001 using one-way ANOVA, Kruskal–Wallis test.

We examined the integrity of the BRB by immunofluorescence staining, labeling blood-retinal barrier GFAP^+^ astrocytes and Müller glia, IB4^+^ ECs, and Cx43^+^ GJs and analyzing their respective immunoreactive areas (Figure 4A). The IB4^+^ immunoreactive area revealed an increase in PBS-treated diabetic mice (10.80 ± 1.44) compared to the ND groups (7.97 ± 1.36, one-way ANOVA *p* < 0.0001). sFKN treatment (6.98 ± 1.10) correlated with decreased IB4^+^ immunoreactivity in the diabetic mice compared to their PBS-treated counterparts (one-way ANOVA *p* < 0.01) (Figure 4B). These data align with our results of diabetes-induced CD31^+^ vascular damage in PBS-treated diabetic mice and associated reduction with sFKN treatment (Figure 2C). In response to diabetes, we observed an increase in GFAP^+^ immunoreactivity in the PBS-treated diabetic mice (20.34 ± 2.81) compared to their ND counterparts (7.81 ± 2.16, one-way ANOVA *p* < 0.01). Administration of sFKN (11.37 ± 2.52, one-way ANOVA *p* < 0.0001) decreased GFAP^+^ immunoreactivity compared to PBS-treated diabetic mice (Figure 4C). Cx43 GJs in PBS-treated diabetic mice (0.82 ± 0.51) were decreased compared to their ND groups (3.40 ± 1.12, one-way ANOVA *p* < 0.01), indicative of gap-junction protein loss (Figure 4D). sFKN (4.58 ± 1.75) increased Cx43 expression in diabetic mice compared to PBS-treated controls (one-way ANOVA *p* < 0.0001). These data suggest that sFKN treatment reversed reactive gliosis and upregulated GJ proteins. To assess the distribution of GJs, we examined Cx43 colocalization to ECs and GFAP astrocytes (Figure S3). Cx43 colocalization to IB4 was abundant in sFKN-treated diabetic mice (10,122 ± 4548) compared to PBS-treated diabetic mice (705.20 ± 332.0, one-way ANOVA *p* < 0.001) (Figure 4E). In addition, in PBS-treated diabetic mice (702.60 ± 399.0), decreased GFAP/Cx43 colocalization was observed compared to PBS-treated ND mice (11,626 ± 3086). We found increased GFAP/Cx43 colocalization in sFKN-treated diabetic mice (10,608 ± 2875, one-way ANOVA *p* < 0.05) compared to their PBS-treated counterparts (Figure 4F).

**Figure 5:**
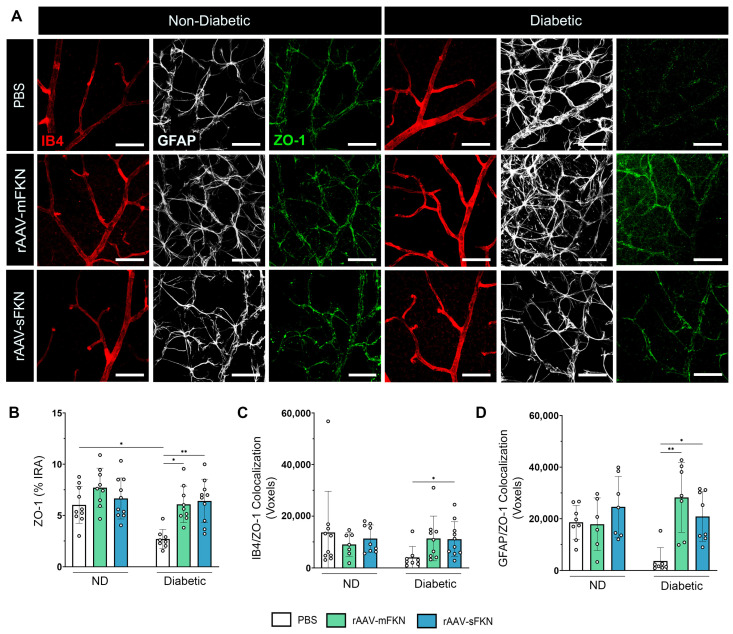
Vascular tight-junction loss is reversed with sFKN in the diabetic retina. (**A**) Confocal images of retinal tissues stained for IB4 (red), GFAP (white), and ZO-1 (green). (**B**) Quantification of retinal immunofluorescence analysis of the ZO-1^+^ immunoreactive area (% IRA). (**C**,**D**) The number of double-positive colocalized pixels (voxels) for IB4/ZO-1 (**C**) and GFAP/ZO-1 (**D**). Data are shown as the mean ± SD, *n* = 7–10 mice per group, where each dot represents an individual mouse. * *p* < 0.05 and ** *p* < 0.01 using one-way ANOVA, Kruskal–Wallis test.

Similarly, we assessed the expression levels of IB4^+^, GFAP^+^, and ZO-1^+^ TJs in the retina (Figure 5A). We found loss of ZO-1 immunoreactivity in PBS-treated diabetic mice (2.69 ± 0.94), indicative of TJ protein loss, compared to PBS-treated ND mice (6.02 ± 1.79, one-way ANOVA *p* < 0.05) (Figure 5B). ZO-1 expression was enhanced with sFKN (6.40 ± 2.12, one-way ANOVA *p* < 0.05) or mFKN (6.07 ± 1.78, one-way ANOVA *p* < 0.01) treatment compared to PBS-treated diabetic mice. These data suggest that FKN treatment, regardless of the isoform, regulated tight-junction protein loss. The distribution of ZO-1 with ECs and astrocytes showed a significant correlation between ZO-1^+^ TJs and IB4^+^ ECs in sFKN-treated diabetic mice (11,048 ± 6816) compared to PBS-treated diabetic mice (4006 ± 4256, one-way ANOVA *p* < 0.05) (Figure 5C and Figure S4). We found increased GFAP/ZO-1 colocalization in sFKN- (20,862 ± 9598, one-way ANOVA *p* < 0.05) and mFKN-treated (28,241 ± 13,521, one-way ANOVA *p* < 0.01) diabetic mice compared to PBS-treated diabetic mice (3599 ± 5202) (Figure 5D).

### 2.6. Visual Acuity Is Enhanced with rAAV-sFKN Treatment

**Figure 6:**
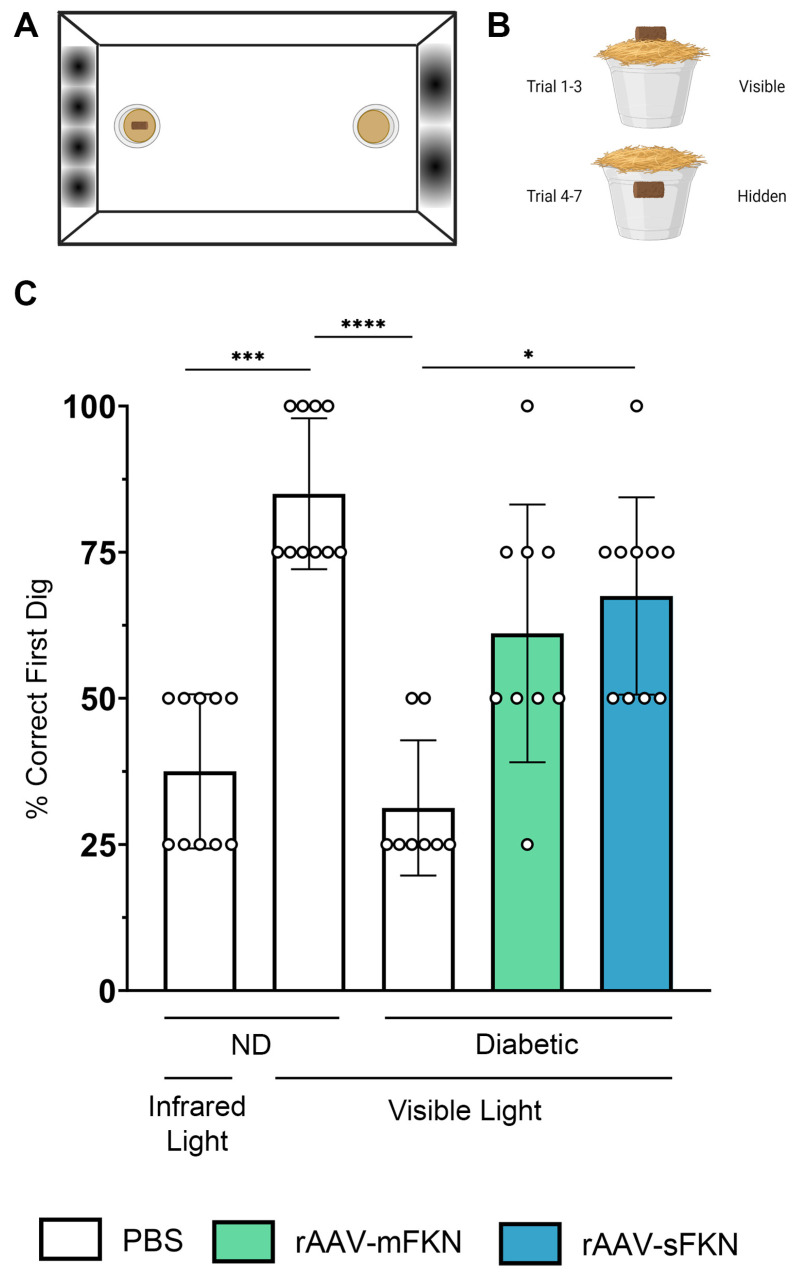
rAAV-sFKN administration restored visual acuity during diabetes. (**A**,**B**) Schematic layout of the visual acuity task displaying distinct sinusoidal spatial frequencies (**A**) and location of the food reward during each trial in the two-choice discrimination task (**B**). (**C**) Percent of correct first dig location. Data are shown as the mean ± SD, *n* = 8–10 mice per group, where each data point represents an individual mouse. * *p* < 0.05, *** *p* < 0.001, and **** *p* < 0.0001 using one-way ANOVA, Kruskal–Wallis test.

We asked if administration of rAAV-sFKN or rAAV-mFKN influenced visual function. A visual acuity assessment using a two-choice discrimination task (Figure 6A,B) was utilized to determine if animals could differentiate between two distinct visual cues. PBS-treated ND animals were tested under visible and infrared light to establish a baseline of visual acuity readouts under sighted and non-sighted conditions, respectively. Under visible light, PBS-treated ND mice discriminated visual cues successfully, reaching levels of performance above 75% (85.0 ± 12.91%); however, under infrared light, these animals found the reward in less than 50% of the trials, showing performance at chance levels (37.50% ± 13.18%, one-way ANOVA *p* < 0.001) (Figure 6C). After establishment of the baseline readout in these mice, we assessed visual acuity in diabetic PBS- (31.25% ± 11.57%), rAAV-mFKN- (61.11% ± 22.05%), and rAAV-sFKN-treated (67.50% ± 16.87%) mice. Diabetic PBS-treated mice showed worse visual acuity, comparable to ND PBS-treated mice in infrared light. Diabetic rAAV-sFKN mice showed poor levels of visual discrimination, similar to the ND control group under visible light conditions. Diabetic rAAV-mFKN mice displayed improved performance compared to diabetic PBS-treated mice, although these results did not reach statistical significance (Figure 6C). These data suggest that treatment with rAAV-sFKN (one-way ANOVA *p* < 0.05) improves visual acuity, further supporting previous findings of reduced vascular damage via regulation of inflammation [12].

## 3. Discussion

### 3.1. Understanding the Role of FKN Isoforms in Microglia and Vascular Function Using rAAV

Our current study reaffirms the significance of the CX3CR1/FKN signaling axis in the regulation of vascular dysfunction through microglial control, employing FKN overexpression via rAAVs. FKN is an efficient modulator of microglial activation that impacts the neurovascular unit. Previous studies have shown that dysregulated CX3CR1/FKN signaling leads to microglial activation, perivascular clustering, proliferation, and increased inflammation in the diabetic retina [12,14,16]. Vascular dysfunction is known to occur at early stages of DR, initiated by the weakening and permeability of the vasculature, promoting reduced retinal blood flow, microaneurysms, and areas of vascular nonperfusion [4]. Treatments for DR primarily focus on inhibitors of inflammatory and vascular growth factors, intra-vitreal steroid injections, or invasive laser eye surgeries. However, these treatments are typically considered when the disease has progressed to its chronic and irreversible stage. Therefore, there is a need to understand the early stages of DR to explore therapeutics that reduce or reverse disease development.

Luxturna, a gene therapy approach employing AAVs has been approved by the United States Food and Drug Administration. Since 2017, Luxturna™ has been used to successfully rectify biallelic mutations in RPE65 [17]. Although AAV therapies are thought to be a promising advancement to prevent vision loss, many risk factors involving undesired immune responses, cytotoxicity, or non-specific transduction have hindered their use.

Our study was inspired by a previous discovery showing that the prophylactic reconstitution of sFKN into the retina of FKN^KO^ mice through rAAVs decreased microglial activation, neuronal loss, inflammation, fibrin(ogen) deposition, and vascular abnormalities and improved visual function. We sought to recapitulate the studies using models with innate FKN expression during diabetes to assess the functional significance of neural-derived FKN overexpression and its influence on microglia and vascular integrity.

Since comparisons of rAAV-GFP versus rAAV-sFKN and rAAV-mFKN have been conducted in several studies [18,19,20], this study addressed the local effect of rAAVs loaded with sFKN or mFKN under non-diabetic versus diabetic conditions. Comparing the effect of the rAAV-sFKN and rAAV-mFKN vectors under non-diseased and diseased conditions is valuable for potential translational applications since it is known that the rAAV-GFP vectors do not exert detrimental effects in the central nervous system.

A primary function of FKN within the CNS is to mitigate microglial pro-inflammatory responses, and numerous studies have highlighted its neuroprotective effects [13,14,21,22,23]. FKN also modulates glia–vascular interaction in diabetic retinas. Microglia-photoreceptor co-cultures revealed strong microglial migratory activity in response to recombinant soluble fractalkine, suggesting that soluble fractalkine released by neurons after injury contribute to the migration of microglia [24]. Intra-vitreal injection of recombinant soluble FKN has been shown to minimize perivascular clustering and fibrin(ogen) deposition in mice lacking FKN and in a model of DR [14]. There is also evidence of reduced pro-inflammatory cytokines and vascular intracellular adhesion molecule-1 (ICAM-1) in diabetic rat models [25]. Furthermore, ex vivo experiments examining the response of retinal microglia to the delivery of exogenous FKN show that the FKN/CX3CR1 signaling pathway influences the vascular diameter [4]. In summary, previous studies have demonstrated that FKN overexpression plays fundamental roles in neurodegeneration, regulating adhesive interaction, and vascular remodeling. Our research supplements these findings by characterizing the distribution of microglia along the vasculature and the expression of gap- and tight-junction proteins in an in vivo mouse model of DR.

### 3.2. FKN Overexpression Alleviates Vascular Damage in the Diabetic Retina

It is accepted that the vasculature of the retina is independent from neurons in directing vascular tone. However, astrocytes and Müller cells dynamically control the function of the vasculature in response to neural activity, conceptually encompassing the neurovascular unit. Leakage of the blood-retinal barrier before glial reactivity suggests that glial cells are early targets of vascular hyperpermeability. Our research indicates that sFKN overexpression by neurons mitigates the extravasation of fibrin(ogen) and vascular damage by alleviating microglial dysregulation in the retinal microenvironment, thereby resolving vascular damage. Since a mutation in the cleavage site of FKN was introduced in the mFKN construct [26], we hypothesize that intracellular pathways within microglia indirectly impact vascular permeability; sFKN can be internalized once it binds CX3CR1, whereas lack of internalization of mFKN can potentially drive different transduction pathways. Hence, the differences in fibrinogen extravasation in sFKN- versus mFKN-treated groups is an area that requires further investigation.

Microglial morphology is often used to measure physiological states in the CNS; thus, we have critically characterized microglial phenotypes in relation to their activation by identifying their ameboid appearance, cellular processes, branching profile in the retina, and homeostatic markers in the CNS and found that rAAV-sFKN administration lessened microglial morphological activation in diabetic mice, comparable to ND controls. Furthermore, investigating the role of FKN via rAAVs beyond the accumulation of fibrin(ogen) by examining “crooked” or “bent” vessels proved to be essential to the comprehension of using a therapeutic model for the treatment of DR.

Microglia in the CNS have an intimate involvement with the vasculature during development, injury, and disease. The data showed that microglia are intimately associated with retinal vasculature upon overexpression of FKN via rAAV therapy, thereby enhancing CX3CR1/FKN signaling and promoting glia–vessel associations and vasoconstrictions in the diabetic retina [4]. Our results revealed an increase in microglia overlapping with retinal vessels upon rAAV-sFKN administration, which decreased the vessel diameter. Overexpression of sFKN to reprogram microglia to a homeostatic state is a potential venue to downregulate their inflammatory status and reduce vascular damage. The functional significance of FKN-mediated amelioration of the vasculature correlated with the improvement in visual acuity in the presence of sFKN and mFKN. We have previously reported that sFKN in FKN^KO^ mice is the preferential isoform in mitigating microglial activation, inflammation, and apoptosis. Currently, these data suggest that sFKN signaling consistently plays a crucial role in the maintenance of normal visual function, correlating with decreased fibrin(ogen) deposition [12].

Activated microglia disrupt the blood-brain barrier via chemokines and cytokine mediated vaso-dilation. These results suggest that dysregulation of the vasculature in diabetic mice is attributed to microglial responses due to insufficient CX3CR1/FKN signaling in the diabetic retina and that overexpression of sFKN can amend EC dysfunction. Significantly more work is needed to confirm microglial involvement in areas exhibiting constriction and the mechanism that alters microglia–vessel and capillary width, pericyte function, and blood flow. The renin–angiotensin system (RAS) has been reported to play a contributing factor in these interactions [4].

### 3.3. FKN Gene Therapy Reverses Tight- and Gap-Junction Loss

Current studies of vascular damage in DR are EC-focused, while our study indicates a role of neuron–microglia interaction in BRB integrity. Astrocytes can strengthen the expression of BRB properties by improving the integrity of EC barriers. We found that astrocyte reactivity in diabetes affects the physiology and function of retinal vascular cells leading to breakdown of the BRB and that rAAV-sFKN treatment decreases astrogliosis [14]. Moreover, GJ and TJ proteins between retinal endothelial cells are the key molecular structures in the maintenance of vascular function. Cx43 GJs are involved in the transfer of small molecules and ions between cells and are ubiquitously prevalent in astrocytes, Müller cells, and endothelial cells, regulating high-glucose-induced retinal EC angiogenesis and retinal neovascularization [27]. ZO-1 TJs are characterized in the inner nuclear layer (INL) and the ganglion cell layer (GCL), ECs, astrocytes, and discrete sites of cell-cell contact, controlling endothelial adherent junctions, angiogenesis, and barrier formation [28,29,30]. In diabetes, impaired Cx43 contributes to the vasomotor decline in diabetic retinas that contributes to the regulation of capillary diameter by pericytes, whereas ZO-1 disruptions have been shown to increase EC permeability [31,32,33]. Upregulation of VEGF signaling leads to the loss of barrier function and TJ integrity in the outer blood-retinal barrier (oBRB), resulting in the leakage of blood contents [34,35]. We have shown that Cx43 and ZO-1 integrity are downregulated in diabetes and ameliorated with response to overexpression of FKN. The relative abundance of these molecules corresponding to astrocyte and ECs correlates with the integrity of these TJ and GJ proteins. However, the mechanism of sFKN overexpression and sustained TJ and GJ integrity remains elusive.

In summary, overexpression of sFKN in the diabetic retina resulted in decreased microglial activation, enhanced vascular integrity, upregulation of gap- and tight-junction proteins, and improved visual acuity. Further research aims to investigate the exact mechanisms that lead to the vasculo-protective effects of sFKN and role in astrocyte–microglia communication. This study provides evidence that rAAVs expressing sFKN reset a homeostatic immune profile in the retina, regulating vascular damage. We showed that sFKN overexpression at early stages of DR can revert microgliosis and BRB alterations, thereby improving visual function, and can be a potential approach to mitigate DR-associated pathology.

## 4. Materials and Methods

### 4.1. Mice

C57BL/6 wild-type (WT) (JAX stock number: 000664, Jackson Laboratory, Bar Harbor, ME, USA; RRID: IMSR_JAX:000664) mice were purchased from The Jackson Laboratory. All experiments used male mice, 6–8 weeks of age, since the dose of streptozotocin used does not induce consistent hyperglycemia in female mice [36,37]. The experiments were performed in accordance with the National Institutes of Health guidelines approved by the UTSA-Institutional Animal Care and Use Committee.

### 4.2. Recombinant Adeno-Associated Viral (rAAV) Vector Production

The vector constructs were engineered, assembled, and produced by Dr. Kevin R. Nash (University of South Florida, Tampa, FL, USA). rAAV constructs were engineered as previously described [18,26]. rAAV serotype 9 vectors, flanked with AAV2 terminal repeat DNA sequences, expressing either mFKN or sFKN were subjected to cloning using PCR on cDNA isolated from mouse brain [26]. rAAV packing of sFKN protein expressed amino acids 1–336. rAAV packing of mFKN DNA comprises all 395 amino acids of full-length FKN protein and contained two mutations (R337A and R338A), preventing cleavage by proteases ADAM10/17 into the soluble form. Vectors of sFKN and mFKN were tagged with hemagglutinin (HA) at the C-terminus for feasible detection of exogenous protein. rAAV particles were quantified using a modified dot plot protocol and are expressed as vector genomes (vg)/mL as described [15,18,20,26]

### 4.3. Two-Hit Inflammatory Streptozotocin (STZ)-Induced Model

To induce hyperglycemia, mice were injected intra-peritoneally (i.p.) once a day for 5 days with 60 mg/kg STZ (Sigma-Aldrich, St. Louis, MO, USA, catalog number: S0130) [14,38]. Control age-matched non-diabetic mice were given citrate buffer as a control. Blood glucose levels were measured weekly for 10 weeks, and animals were considered to be hyperglycemic when blood glucose levels were >250 mg/dL. Due to the reoccurring infections diabetic patients experience, lipopolysaccharide (LPS) intra-peritoneal injections were conducted to mimic low-grade systemic inflammation and relative increased plasma levels [23,39,40]. Acute LPS administration can cause hypoglycemia, inducing variation in circulating glucose levels in diabetic mice [41]. To prevent masking hyperglycemia, diabetic mice received one injection of 0.08 mg/kg LPS per week for two weeks prior to euthanasia.

### 4.4. Intra-Vitreal Injection with rAAVs

Four weeks after hyperglycemic induction, mice were therapeutically treated with rAAVs expressing mFKN or sFKN. Medical heated underpads were placed on the table and turned on prior to the start of the injection to maintain optimal warmth for the animal. The microinjector was prepared under a dissecting microscope using a 31G beveled Nanofil needle and syringe attached to a Micro 4TM Microsyringe Pump Controller and foot switch (World Precision Instruments, Sarasota, FL, USA) for accurate injection volume. The 10 μL syringe was filled with rAAVs expressing mFKN or sFKN at a concentration of 1 × 10^12^ vg/mL, diluted in 1x PBS (Cytiva, Marlborough, MA, USA, catalog number: SH30258.02) or PBS for those in the control group. Before the commencement of the procedure, we confirmed that the appropriate volume was dispensed from the bevel of the needle. Mice were anesthetized with 5% isoflurane in oxygen and then placed on the heated underpad. Before injections, we confirmed that the mouse was adequately anesthetized by testing the pedal withdrawal reflex before disinfecting the right orbital with a 70% alcohol prep pad (VWR, Radnor, PA, USA, catalog number: 75856-902). The tip of the needle was placed at the limbus of the eye. With the bevel facing up and positioned at a 45° angle, the eye was punctured and the needle was inserted (~0.5 mm). The animal received a single intra-vitreal injection of rAAV-sFKN or rAAV-mFKN (200 nL/s for 5 s). The needle was left in place for 5 s to limit backflow after the infusion, then removed slowly. Before returning to the housing cage, one drop of proparacaine hydrochloride ophthalmic solution USP, 0.5% (Alcon, Geneva, Switzerland, catalog number: H14233-0216) was applied to the eye as a topical antibiotic. Mice were observed for 24 h to ensure no signs of distress (Figure 1A).

### 4.5. Tissue Collection

Mice were transcardially perfused with cold 1x Hank’s balanced salt solution (HBSS, Lowell, MA, USA; Cytiva, catalog number: SH30588.02). Eyes were enucleated and placed in 4% paraformaldehyde (PFA) for 20 min. Retinas were dissected out of the globe of the eye and placed in 1% PFA for 1 h. Fixed retinas were then placed in cryoprotection solution (200 mL glycerol, 200 mL 0.4M Sorenson’s buffer, and 600 mL MilliQ water) overnight at 4 °C. The following day, the retinas were transferred in cryostorage solution (500 mL 0.2M PO4, 10 g PVP-40, 300 g sucrose, and 300 mL ethylene glycol) and stored at −20 °C.

### 4.6. Immunofluorescence Staining

Retinas were divided from the center of the optic disk to the peripheral edges into ¼ leaflets chosen at random to visualize proteins of interest. Cryostorage solution was rinsed with 1x PBS. Prior to blocking the tissues, the retinal leaflets were blocked overnight in 10% goat serum containing 1% Triton-X 100 at 4 °C for immunohistochemical analysis. Next, tissues were incubated overnight at 4 °C with primary antibodies diluted in blocking solution (10% goat serum (RRID: 2336990) containing 1% Triton-X 100) to visualize proteins of interest, rabbit anti-ionized calcium binding adaptor molecule-1 (Iba1) (RRID: AB_839504), mouse anti-neuronal nuclei (NeuN) (RRID: AB_2298772), mouse anti-RNA binding protein, mRNA processing factor (RBPMS) (RRID: AB_2687403), rat anti-glial fibrillary acidic protein (GFAP) (RRID: AB_2532994), rat anti-pecam-1 (CD31) (RRID: AB_393571), rabbit anti-fibrinogen (RRID: AB_2894406), biotin isolectin B4 (IB4) (RRID: AB_2314661), rabbit anti-connexin 43 (Cx43) (RRID: AB_476857), and rabbit anti-zonula occudens-1 (ZO-1) (RRID: AB_2533456) as outlined in Supplementary Table S1. Tissues were washed 9x for 5 min each with 0.1% Triton-X in 1x PBS to remove unbound antibodies. Tissues were incubated for three hours in species-specific secondary antibodies to visualize proteins of interest followed by 9 washes each at 5 min in PBS with 0.1% Triton-X 100. Next, tissues were incubated in Hoechst 3342 (ThermoFisher Scientific, Waltham, MA, USA, catalog number: H1399) for 7 min to label cellular nuclei, followed by 3 washes in PBS for 5 min each. Tissues were mounted on Superfrost Plus microscope slides (ThermoFisher Scientific, catalog number: 12-550-15) and cover slipped using Fluorsave (Sigma-Aldrich, catalog number: 345789). Antibody combinations and species-specific primary and secondary antibody combinations are outlined in Supplementary Table S2.

### 4.7. Confocal Microscopy

Confocal microscopy was employed with a Zeiss 710 NLO confocal microscope. Imaging was performed of the vascular plexus including the retinal ganglion cell layer. Then, 3D compositions of confocal images were generated using Imaris software v7.2 (Bitplane, Belfast, UK). Six images were obtained per ¼ retinal leaflet, namely, 2 images at the central retina nearest the optic nerve, 2 images in the middle of the leaflet, and 2 images in the outer leaflet, per mouse. Quantifications shown in graphical figures represent the average of the six images taken per mouse. Manual counts were conducted to quantify Iba1^+^ microglial and NeuN^+^RBPMS^+^ neuronal cell body densities; cells were manually counted in 40x images using the counter tool in Adobe Photoshop version 21.0.3. The percent immunoreactive areas of CD31^+^ and IB4^+^ endothelial cells, GFAP^+^ glia, fibrinogen, ZO-1^+^ tight junctions, and Cx43^+^ gap junctions were quantified by uploading the Imaris-processed confocal images and converting to 32-bit in ImageJ Fiji analysis software (NIH), and an automatic threshold was applied. The data were normalized by volume based on the X, Y, and Z coordinates (i.e., 212 µm × 212 µm × Z-stack thickness) to account for changes in the confocal Z-stack thickness and image size (scale settings—distance in pixels: 1024; known distance: 206.25).

### 4.8. Sholl Analysis and Schoenen Ramification Index Analysis

Iba1 immunoreactivity alone is not a sensitive marker for evaluating activation of microglia [42]. Therefore, to accurately analyze the level of microglial morphological activation three forms of analyses to determine the morphological changes in microglia were conducted in a sequential manner: (1) Sholl analysis, (2) Schoenen ramification index (RI) determination, and (3) transformation index (TI) determination. Iba1 staining was analyzed using the neuroanatomy plugin following the ImageJ protocol [43,44]. Images were converted to 8-bit image type, followed by applying and adjusting the contrast to best visualize the branches of the microglia. For the best and optimal readouts, parameters were implemented to eliminate confounding variables. Only cells with major branches were included in the analysis; we excluded cells that touched a border of the Z-stack or those cells that were closer to the border or connected to each other. These parameters were used to randomly select single microglia for the analysis. A radius was drawn to indicate the region of interest (ROI), from the center of the cell to the end of the longest branch to set the upper and lower limit for concentric circle placement. The contrast and threshold were adjusted, followed by the clear background function to remove background noise. Using the Neuroanatomy plugin, we applied the best-fit parameters. The distance between each circle was set at 5 µm centered on the soma and increasing 2 μm with every circle. The number of times that the microglial branches intercepted each of the circles was calculated in every image (1 microglia per 6 images per mouse). Coordinates from the Sholl plot were taken and averaged. The data generated from the output included the process maximum (N_m_, the maximum number of intersections for the cell), the critical value (C_r_, the distance from the soma where N_m_ occurred), the maximum branch length (μm, the maximum radius at which a branch intersection occurred), the number of primary branches (N_p_, the number of branches that originated from the microglial soma), and the centroid value (calculated coordinates from the cell soma). These parameters gave us the calculated values for the Schoenen RI (N_m_/N_p_), quantifying cell branching density ranging from 0 to 15, with an RI value closer to 15 representing highly branching, ramified microglia with long cellular processes. Low RI values represent retracted microglia with minimal branching of cellular processes [45,46].

### 4.9. Transformation Index Analysis

Microglial morphological changes were further determined by the transformation index (TI). The same cells that were isolated for the Sholl and Schoenen analysis were analyzed in ImageJ. Tracing was used to determine the perimeter and area of a microglia. The TI was calculated using the following equation: perimeter^2^/4π × area^2^ [13,47]. The TI values are expressed as a range from 1 to 100, with a TI value closer to 1 representing a circular, amoeboid microglia with fewer and/or shorter cellular processes. Higher TI values represent ramified microglia with extensive branching and smaller cell bodies.

### 4.10. Retinal Tortuosity Analysis

CD31^+^ endothelial cell staining of retinal wholemounts was analyzed using ImageJ software. Vessels containing 3 or more junctions/segments arising from the main arteriole were traced with straight and segmented lines spanning the most lateral points of the vessel path (Figure 3D). The resulting paths were used for tortuosity index (ToI) calculation length of a segmented line/distance between the start and end. The integrated curvature (IC) was calculated by taking the sum of the angles between the line segments and dividing by the distance between the start and end point. A linear vessel has a ToI value closer to 0, whereas a curved, sinuous vessel has a ToI value closer to 2.5. A straight vessel has an IC value of 0, and an exaggeratedly angled vessel has an IC value closer to 1. ToI values and IC values (representing degree over distance) were normalized against the average values from nondiabetic mice treated with PBS [48]. Six images were obtained per ¼ retinal leaflet per mouse, and two randomly selected arteries were analyzed and averaged for each image.

### 4.11. Microglial and Vessel Interaction Analysis

To determine the overlap between Iba1^+^ microglia and IB4^+^ endothelial cells, we used the Imaris colocalization tool to determine the interactions of the microglia along the blood vessels in the image using their respective fluorochromes (Supplementary Table S2). Briefly, the two channels of interest were selected, and an automatic threshold using the One Time Point setting (to calculate the intensity of the fluorochrome histogram for the entire 3D composition) was performed on both channels. Imaris colocalization volume statistics were used. The data were graphed as the number of total colocalized voxels between two channels. A colocalization tool was applied to GFAP and ZO1, IB4 and ZO1, GFAP and Cx43, and IB4 and Cx43 to examine and confirm the abundance of these proteins with their respective antibodies (Supplementary Table S2). To further characterize microglial interaction and capillary width, ImageJ software was used to measure contacts (within 0.40 µm) between microglia (Iba1^+^) and blood capillaries (IB4^+^) (m+) as well as capillary areas devoid of microglial contact (m–). We defined capillaries as IB4^+^ vessel structures with a diameter of 7 μm or less [49]. To measure capillary width, six images were obtained per ¼ retinal leaflet per mouse, and two randomly selected capillary areas with and without microglia-capillary contact were analyzed and averaged on each image.

### 4.12. Flow Cytometry of Blood and CNS Tissues

Peripheral blood was drawn by submandibular vein puncture and collected in 30 μL of 5000 U/mL heparin (30 μL of heparin per 500 μL of blood) (Sigma-Aldrich, catalog number: H3393) in 1.5 mL Eppendorf tubes. Lysing of blood was conducted under hypotonic conditions as previously in water for 20 s followed by the addition of 10x HBSS and 1x HBSS (supplemented with 10 mM HEPES) and centrifuged at 4 °C for 7 min at 2200 rpm. Brain and spinal cord tissues were isolated and homogenized in Gibco RPMI 1640 media (ThermoFisher Scientific, catalog number: 32404014) and resuspended in 10 mM HBSS/HEPES, followed by the isolation of mononuclear cells using Percoll gradients [50,51]. Cell suspensions were prepared in cell staining buffer (Biolegend, San Diego, CA, USA, catalog number: 420201). Cells were blocked with Fc block (BD Pharmingen, Franklin Lakes, NJ, USA, catalog number: 553142) for 15 min. Blood and CNS tissues were subjected to staining with an antibody cocktail labeling peripheral leukocytes and CNS mononuclear cells with antibodies against CD11b, CD45, Ly6C, and CD11c (Supplementary Table S3) and adding Zombie Aqua as a viability dye. Mononuclear cells from brain and spinal cord tissues were analyzed by flow cytometry using a mix of antibodies to identify monocyte-derived inflammatory microglia (CD45^Lo^CD11b^+^Ly6C^+^) and homeostatic microglia (CD45^Lo^CD11b^+^P2RY12^+^). For peripheral immune cells, non-classical monocytes were characterized as CD45^Hi^CD11b^+^Ly6C^−^, classical monocytes as CD45^Hi^CD11b^+^Ly6C^+^, myeloid dendritic cells as CD45^Hi^CD11b^+^CD11c^+^, and conventional dendritic cells as CD45^Hi^CD11b^−^CD11c^+^. Cell populations were identified using an LSR-II cytometer (BD Bioscience; RRID: SCR_002159) and analyzed using FlowJo software v9.2; RRID: SCR_008520).

### 4.13. Visual Acuity Test

Mice were housed individually and shaped to engage in digging behavior to obtain a food reward. Each day, they were presented with a reward cup with a capacity of 30 mL, which contained 1–2 g of a food pellet and a small chocolate cereal crumb reward (Cocoa Krispies, Kellogg’s). These rewards were strategically buried beneath fine-grain wood chip bedding (Sani-Chip IRR, LabSupply, Durham, NC, USA) mixed with cumin to eliminate any olfactory cues. Following a period of food deprivation, during which the mice’s weight was reduced to 80–85% of their ad libitum weight, they were then situated within a rectangular plexiglass apparatus measuring 14 inches by 10 inches, as previously detailed (Figure 6A) [52]. The chamber featured one short wall with 4 diffuse black stripes, while the opposite wall had 2 diffuse black stripes [12]. Two plastic medicine cups containing cumin-scented wood chip bedding were embedded in opposite sides of the chamber floor along the visual cues before placing the mice in the apparatus [53]. A high-value chocolate cereal crumb reward was always placed in the cup next to the wall with 4 stripes. Each animal completed a total of 7 trials. For the first three trials, the reward was visibly placed on top of the bedding to allow the mice to learn the reward location and associate it with the visual cue. For the remaining four trials, the reward was consistently buried 2 cm beneath the cumin-scented bedding. The location of the first dig was used as a measure of performance and plotted as percent of correct digs during testing trials (Trials 4–7). During training and testing, the apparatus was surrounded by a black curtain and rotated 90° clockwise every trial orientation to prevent the use of external visual cues to guide performance. Additionally, all experiments were conducted in the presence of a white noise generator to prevent animals from using external auditory cues to find the reward. Mice were left undisturbed in the apparatus until a choice (correct or incorrect) was made. If no digging occurred after 3 min, the animal was removed from the apparatus, and the trial was counted as incorrect. The apparatus was cleaned with 70% ethanol to eliminate odor traces after the completion of each trial. To simulate conditions of blindness, control sighted mice were tested under infrared light in 7 additional trials, following normal light visible trials.

### 4.14. Statistical Analysis

All analyses were conducted using GraphPad Prism v10.0, and a *p* value < 0.05 was considered statistically significant. Statistical significance is denoted as * *p* value < 0.05, ** *p* value < 0.01, *** *p* value < 0.001, and **** *p* value < 0.0001. Statistical tests performed included a one-way ANOVA with Kruskal–Wallis test, using the treatment type as the variable. Within the diseased groups, comparisons across the treatments were selected and analyzed using the unpaired Student’s *t* test with Welch’s correction. All quantitative image assessments were performed by a scientist blind to the experimental conditions.

## Data Availability

The datasets used and/or analyzed during the current study are available from the corresponding author on reasonable request.

## Supplementary Materials

The following supporting information can be downloaded at: https://www.mdpi.com/article/10.3390/ijms25031727/s1.
