# Supplementary material for: Therapeutic Delivery of Soluble Fractalkine Ameliorates Vascular Dysfunction in the Diabetic Retina
Source: Int J Mol Sci. 2024 Jan 31;25(3):1727. doi: 10.3390/ijms25031727 (PMC10855319; doi:10.3390/ijms25031727)
Supplement: Supplementary file 1 [file ijms-25-01727-s001.zip › ijms-2800270-supplementary.pdf]

Supplementary Figures and Figure Legends

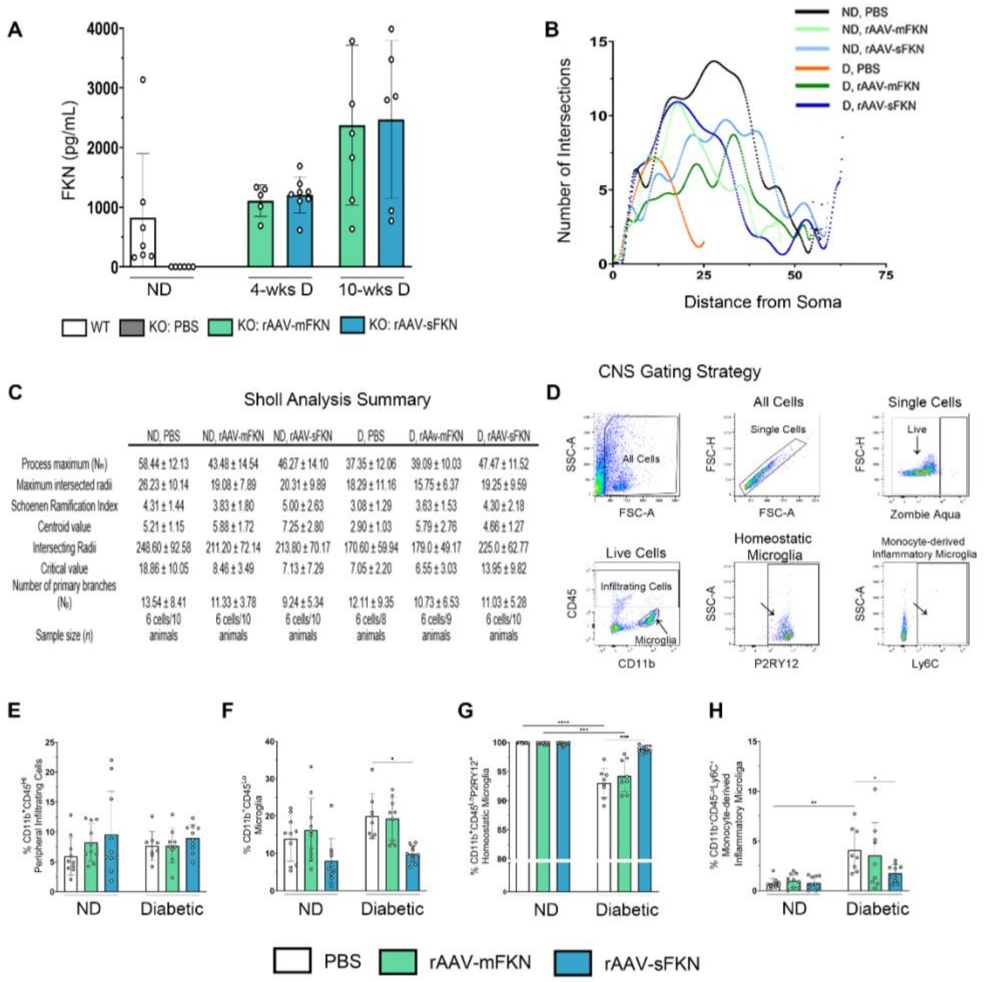

	ND, PBS	ND, rAAV-mFKN	ND, rAAV-sFKN	D, PBS	D, rAAv-mFKN	D, rAAV-sFKN
Process maximum (N_m)	58.44 ± 12.13	43.48 ± 14.54	46.27 ± 14.10	37.35 ± 12.06	39.09 ± 10.03	47.47 ± 11.52
Maximum intersected radii	26.23 ± 10.14	19.08 ± 7.89	20.31 ± 9.89	18.29 ± 11.16	15.75 ± 6.37	19.25 ± 9.59
Schoenen Ramification Index	4.31 ± 1.44	3.83 ± 1.80	5.00 ± 2.63	3.08 ± 1.29	3.63 ± 1.53	4.30 ± 2.18
Centroid value	5.21 ± 1.15	5.88 ± 1.72	7.25 ± 2.80	2.90 ± 1.03	5.79 ± 2.76	4.66 ± 1.27
Intersecting Radii	248.60 ± 92.58	211.20 ± 72.14	213.80 ± 70.17	170.60 ± 59.94	179.0 ± 49.17	225.0 ± 62.77
Critical value	18.86 ± 10.05	8.46 ± 3.49	7.13 ± 7.29	7.05 ± 2.20	6.55 ± 3.03	13.95 ± 9.82
Number of primary branches (N_p)	13.54 ± 8.41	11.33 ± 3.78	9.24 ± 5.34	12.11 ± 9.35	10.73 ± 6.53	11.03 ± 5.28
Sample size (*n*)	6 cells/10 animals	6 cells/10 animals	6 cells/10 animals	6 cells/8 animals	6 cells/9 animals	6 cells/10 animals

Figure S1. rAAV-sFKN shifts microglia towards a homeostatic phenotype. (**A**) Quantification of FKN (pg/mL) by ELISA in ND wild-type (WT) and PBS-treated FKN^{KO}, 4-weeks diabetic and 10-weeks diabetic mice treated with rAAV-mFKN (green bars) or rAAV-sFKN (blue bars). This data shows the characterization of the diabetic model using FKN^{KO} mice and rAAV expressing mFKN or sFKN, currently under peer-review in another peer review journal. (**B-C**) Microglia homeostatic morphology in the retina is noted by extended processes intersecting successive concentric circles relative to the distance of the cell body was represented in a Sholl analysis plot (**B**) and Sholl analysis data summary (**C**). (**D**) Gating strategy to identify $CD11b^+CD45^{Lo}$ microglia in brain and spinal cord tissues, $CD11b^+CD45^{Hi}$ infiltrating cells, $CD11b^+CD45^{Lo}P2RY12^+$ homeostatic microglia, and $CD11b^+CD45^{Lo}Ly6C^+$ monocyte-derived inflammatory microglia. (**E-H**) Graphical representation of flow cytometric quantification of $CD11b^+CD45^{Hi}$ infiltrating cells in brain and spinal cord tissues (**E**), $CD11b^+CD45^{Lo}$ microglia (**F**), $CD11b^+CD45^{Lo}P2RY12^+$ homeostatic microglia (**G**), and $CD11b^+CD45^{Lo}Ly6C^+$ monocyte-derived inflammatory microglia (**H**). Data is shown as mean ± SD, *n* = 8-10 mice per group where each data point represents an individual mouse. *p < 0.05, **p < 0.01, ***p < 0.001 and ****p < 0.0001 using one-way ANOVA, Kruskal-Wallis

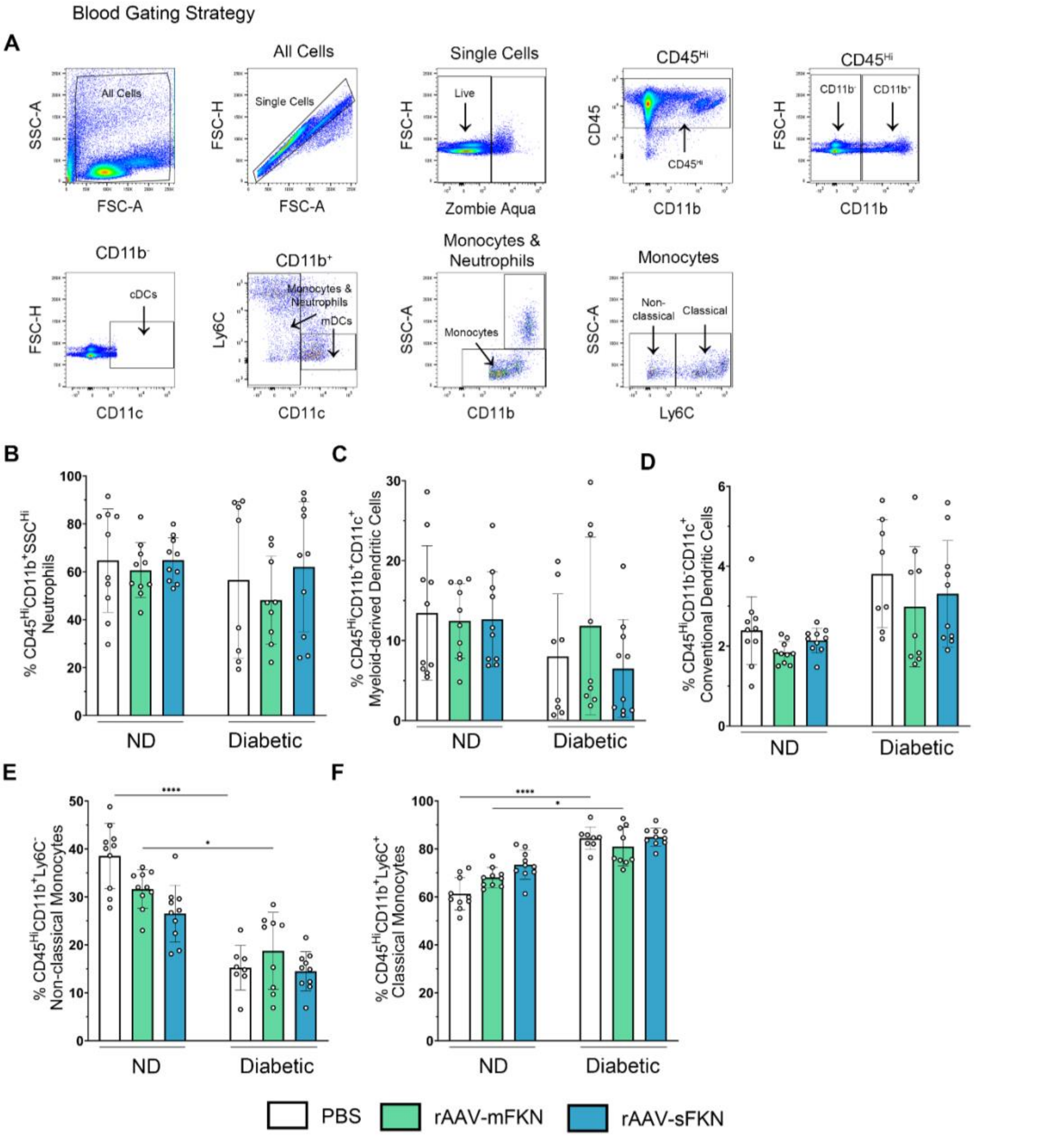

Figure S2. rAAVs do not alter the peripheral immune cells in blood tissue. (**A**) Gating strategy to identify CD45^{Hi}CD11b^{+}SSC^{Hi} neutrophils, CD45^{Hi}CD11b^{+}Ly6C$^{–}$ non-classical monocytes, CD45^{Hi}CD11b^{+}Ly6C^{+} classical monocytes, CD45^{Hi}CD11b^{+}CD11c^{+} myeloid-derived dendritic cells, and CD45^{Hi}CD11b$^{–}$CD11c^{+} conventional dendritic cells in blood leukocytes. (**B-E**) Graphical representation of flow cytometric quantification of CD45^{Hi}CD11b^{+}SSC^{Hi} neutrophils (**B**), CD45^{Hi}CD11b^{+}CD11c^{+} myeloid-derived dendritic cells (**C**), CD45^{Hi}CD11b$^{–}$CD11c^{+} conventional dendritic cells (**D**), CD45^{Hi}CD11b^{+}Ly6C$^{–}$ non-classical monocytes (**E**), CD45^{Hi}CD11b^{+}Ly6C^{+} classical monocytes (**F**). Data is shown as mean ± SD, n = 8-10 mice per group where each data point represents an individual mouse. *$p < 0.05$ and ****$p < 0.0001$ using one-way ANOVA, Kruskal-Wallis test.

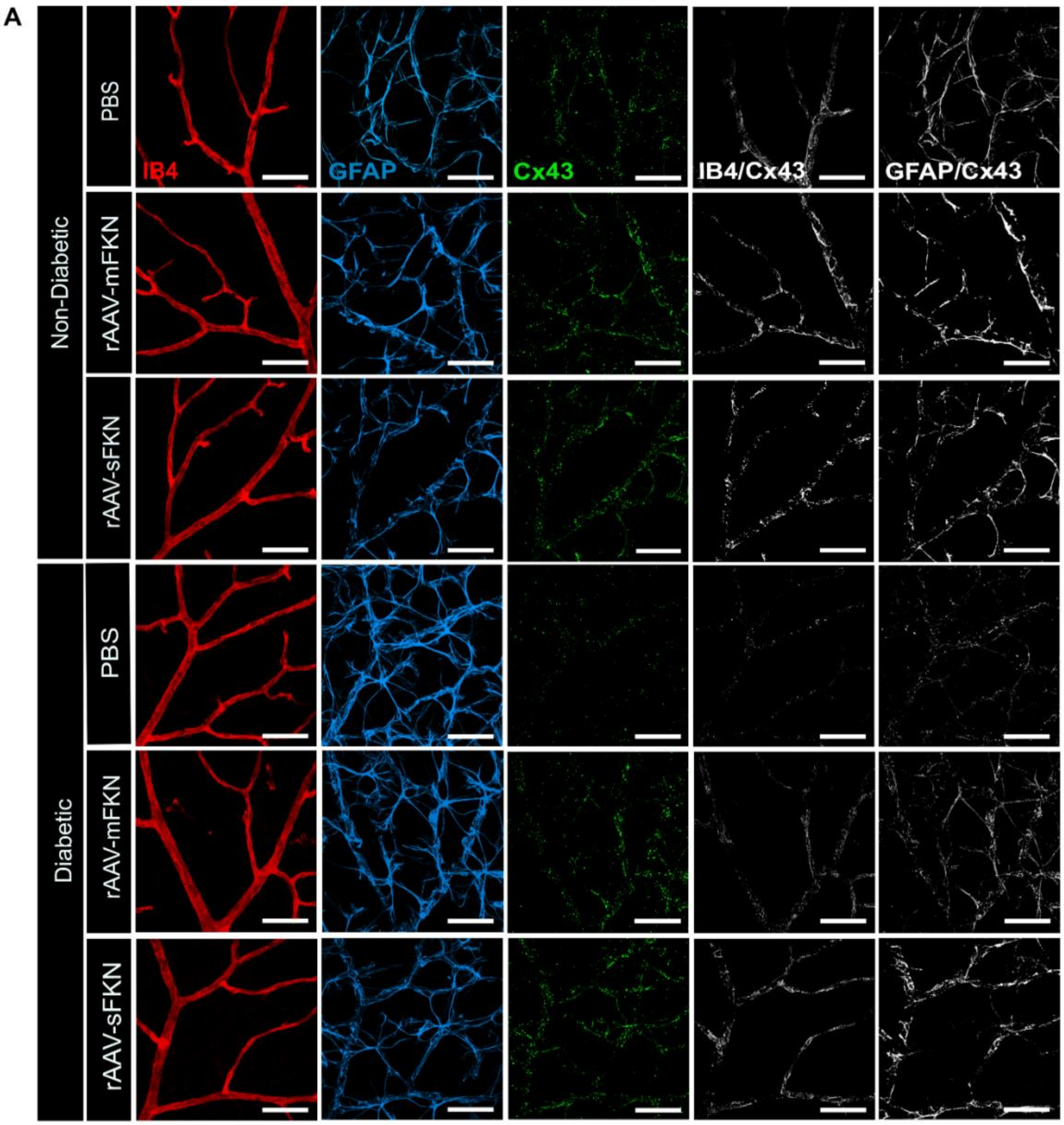

Figure S3. Gap-junction distribution in endothelial cells and astrocytes is increased with rAAV-sFKN. (**A**) Confocal images of retinal tissues stained for IB4 (red), GFAP (blue), Cx43 (green), and double immunofluorescence colocalized regions for IB4/Cx43 and GFAP/Cx43 (white) using Imaris software.

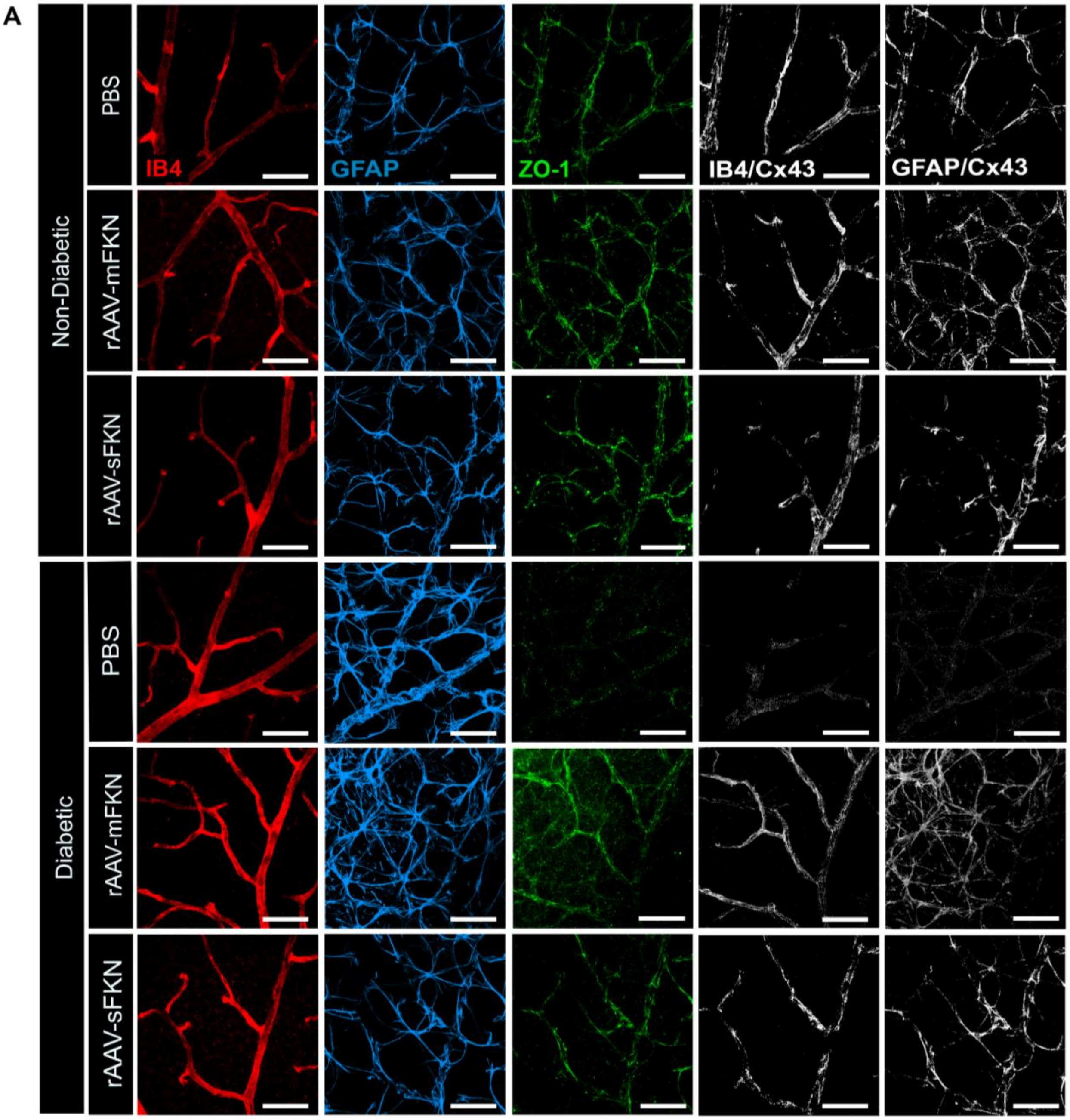

Figure S4. sFKN or mFKN treatment contributes to increased tight-junction distribution in endothelial cells and astrocytes. (**A**) Confocal images of retinal tissues stained for IB4 (red), GFAP (blue), ZO-1 (green), and double immunofluorescence colocalized regions for IB4/ZO-1 and GFAP/ZO-1 (white) using Imaris software.

Supplementary Tables

Supplementary Table 1. Antibodies for immunohistochemistry analysis

Target antigen (Clone)	Company	RRID	Dilution
**Primary antibodies**			
Rabbit anti-ionized calcium binding adaptor molecule-1 (Iba1)	FUJIFILM-Wako	AB_839504	1:3000
Mouse anti-neuronal nuclei (NeuN)	Millipore	AB_2298772	1:4000
Guinea Pig anti RNA-binding protein with multiple splicing (RBPMS)		AB_2687403	1:500
Rat anti-glial fibrillary acidic protein (GFAP)	Invitrogen	AB_2532994	1:4000
Biotinylated Griffonia Simplicifolia Lectin I (GSL I) Isolectin B4 (IB4)	Vector Laboratories	AB_2314661	1:100
Rabbit anti-fibrinogen	Agilent	AB_578481	1:2000
Rabbit anti-zonula occludens-1 (ZO-1)	Invitrogen	AB_2533456	1:500
Rabbit anti-connexin 43 (Cx43)	Sigma-Aldrich	AB_476857	1:1000
Rat anti-platelet endothelial cell adhesion molecule (PECAM-1/CD31)	BD Biosciences	AB_393571	1:500
**Secondary antibodies**			
Goat anti-mouse Cy3	Jackson ImmunoResearch Laboratories, Inc	AB_2338709	1:1000
Goat anti-rabbit 488		AB_2338058	1:1000
Donkey anti-guinea pig Cy3		AB_2340460	1:1000
Goat anti-rat AlexaFluor 647		AB_2338394	1:1000
Goat anti-rabbit Cy3		AB_2338006	1:1000
Donkey anti-rat Alexa Fluor 488		AB_2340684	1:1000
Streptavidin Cy5		AB_2337245	1:1000

Supplementary Table 2. Combination of antibodies used for analysis

Target antigen	Primary antibody	Secondary antibody
Iba1	Rabbit anti-Iba1	Goat anti-rabbit 488
NeuN	Mouse anti-NeuN	Goat anti-mouse Cy3
RBPMS	Mouse anti-RBPMS	Donkey anti-guinea pig Cy3
GFAP	Rat anti-GFAP	Dokney anti-rat AlexaFluor 488
CD31	Rat anti-CD31	Goat anti-rat AlexaFluor 647
Fibrinogen	Rabbit anti-fibrinogen	Goat anti-rabbit Cy3
IB4	Biotin IB4	Streptavidin Cy5
ZO-1	Rabbit anti-ZO-1	Goat anti-rabbit Cy3
Cx43	Rabbit anti-connexin 43	Goat anti-rabbit Cy3

Supplementary Table 3. Antibodies for flow cytometry analysis

Target antigen (Clone)	Company	RRID	Dilution
CD11b PE-CF594 (M1/70)	BD Bioscience	AB_11154422	1:100
CD45 Pacific Blue (30-F11)	Invitrogen	AB_1518806	1:100
CD11c PE-Cy7 (N418)		AB_465552	1:50
P2RY12 APC (S16007D)	Biolegend	AB_2721469	1:100
Ly6C PE (HK1.4)		AB_1186132	1:30
